# Water bath is more efficient than hot air oven at thermal inactivation of coronavirus

**DOI:** 10.1186/s12985-023-02038-7

**Published:** 2023-05-02

**Authors:** Xinxia Gu, Ting Cao, Jun Mou, Jie Liu

**Affiliations:** Laboratory of Infectious Diseases and Vaccine, West China School of Medicine, West China Hospital, Sichuan University, 88 Keyuan S. Rd, Chengdu, 610041 China

**Keywords:** Virus, Infectivity, Biosafety, Heat transfer, Specific heat capacity, Heating rate

## Abstract

**Background:**

Thermal inactivation is a conventional and effective method of eliminating the infectivity of pathogens from specimens in clinical and biological laboratories, and reducing the risk of occupational exposure and environmental contamination. During the COVID-19 pandemic, specimens from patients and potentially infected individuals were heat treated and processed under BSL-2 conditions in a safe, cost-effective, and timely manner. The temperature and duration of heat treatment are optimized and standardized in the protocol according to the susceptibility of the pathogen and the impact on the integrity of the specimens, but the heating device is often undefined. Devices and medium transferring the thermal energy vary in heating rate, specific heat capacity, and conductivity, resulting in variations in efficiency and inactivation outcome that may compromise biosafety and downstream biological assays.

**Methods:**

We evaluated the water bath and hot air oven in terms of pathogen inactivation efficiency, which are the most commonly used inactivation devices in hospitals and biological laboratories. By evaluating the temperature equilibrium and viral titer elimination under various conditions, we studied the devices and their inactivation outcomes under identical treatment protocol, and to analyzed the factors, such as energy conductivity, specific heat capacity, and heating rate, underlying the inactivation efficiencies.

**Results:**

We compared thermal inactivation of coronavirus using different devices, and have found that the water bath was more efficient at reducing infectivity, with higher heat transfer and thermal equilibration than a forced hot air oven. In addition to the efficiency, the water bath showed relative consistency in temperature equilibration of samples of different volumes, reduced the need for prolonged heating, and eliminated the risk of pathogen spread by forced airflow.

**Conclusions:**

Our data support the proposal to define the heating device in the thermal inactivation protocol and in the specimen management policy.

## Background

Specimen from patients and healthy volunteers often carry, or are contaminated with, known and unknown pathogens. A large number of victims acquired hepatitis and immunodeficiency syndrome through contaminated needle injections, blood transfusions, or dialysis before the implementation of mandatory screening for the respective viral pathogens in medical practices [1, 2]. Laboratory staffs who processing specimens occupationally became exposed to infectious agents and acquired infections through accidental contact with pathogenic bacteria, viruses, parasites, and fungi [3, 4]. The pathogens in medical waste without inactivation or sterilization could persist in the environment for an extended period of time and posed a threat to public health [5, 6]. During the pandemic of COVID-19, WHO and other authorities established guidelines for the processing of specimens and disposal of medical wastes to reduce the risk of exposure of hospital staff and the public to SARS-CoV-2 [7, 8]. Inactivation of specimens has been a mandatory procedure to protect hospital staff and the environment from contamination and spread of pathogens.

Various methods are recommended to inactivate pathogens in specimens, including chemical and physical, depending on the convenience, effectiveness, and compatibility with downstream biological processing. For example, formaldehyde was commonly used to eliminate pathogen infectivity while preserving cell morphology, which was often recommended for pathological and morphological diagnostics [9]. However, formaldehyde potentially altered protein structure, and was not recommended for diagnostic purposes depending on the nature of protein [10]. Detergents and Trizol were commonly used for inactivation and nucleic acid extraction, but had to be removed from samples to minimize the impact on downstream processing [11]. Chemical inactivation, such as fixation and denaturation, was not preferred for samples for immunological and nucleic acid based diagnostics because of the potential alteration of the antigenic epitopes critical for immunoassays and fragmentation of nucleic acids for polymerase chain reaction [12]. Physical inactivation mainly uses radiation and thermal energy to eliminate pathogen infectivity. Ultraviolet radiation has been used to inactivate viruses by altering viral protein structure and nucleic acids, but required considerable protection from occupational exposure, optimization of dose and exposure time [13], and the effectiveness was negated by the presence of protein in the sample [14]. Gamma irradiation has been used to inactivate a variety of pathogens. However, the accessibility, cost, and potential radioactive hazard limit its application. Thermal inactivation is the most convenient, cost-effective, and effective approach applicable to a variety of samples, such as plasma, urine, feces, nasopharyngeal, and oropharyngeal samples for serological, immunological and biochemical analysis and pathogen identification. Thermal energy denatures nucleic acids and randomly alters the structure and conformation of proteins, preventing attachment, fusion, and replication of pathogens and impairing the infectivity [15, 16], and has been used to inactivate various pathogens including viruses, bacteria, and fungi [17–19]. Thermal energy can also inactivate enzymes, denature protein structure, alter bioactivity, and oxidize molecules critical to diagnostics. Excessive energy could lead to false test results, particularly in samples with trace amounts of target biomarkers [20]. Although it is the most common approach to reduce pathogen infectivity with limited impact on the sample [15, 21, 22], thermal inactivation needs to be optimized to balance pathogen inactivation and sample integrity.

The Optimal temperature and duration of heat exposure depend on the intrinsic properties of the pathogen, the nature of the sample, and the downstream biological assay. For example, the titer of Middle East Respiratory Syndrome virus diminished by 4log10 in 25 min at 56℃. However, at 65℃, this decrease was achieved within 1 min [23]. The titer of SARS coronavirus diminished by 4log10 at 56 °C in 15 min [14]. Above 95 °C, the SARS-CoV-2 RNA copies and detection rate decreased significantly [24]. Prolonged exposure to excessive thermal treatment also altered the diagnostic assessment of antibodies [18, 25] and RNAs [21]. Optimized temperature and minimized exposure to heat reduce the adverse effects on samples and ensure the accuracy of assays. The WHO recommended 56 °C for 30 min for SARS-Cov-2 inactivation [7, 26]. This combination of temperature and duration has been adapted and proven safe without significantly affecting biological markers or viral nucleic acid detection [18, 24]. The efficiency of thermal inactivation also depended on the heating device and thermal energy transfer medium, which had different heating rates, thermal conductivity, and specific heat capacities [27]. However, these key factors are often neglected or not defined in most inactivation protocols and guidelines. For example the World Health Organization (WHO), the Centers for Disease Control and Prevention (CDC, USA), and the Chinese Center for Disease Control and Prevention did not define heating devices in their guidelines for COVID-19 specimen inactivation [7, 28]. To investigate how the heating device, heat transfer medium, and specimen container affected the efficiency of pathogen inactivation, we compared the inactivation efficiency of water bath and forced-air oven that have been commonly used in hospitals and biological laboratories, using transmissible gastroenteritis virus of swine (TGEV) as a model pathogen. TGEV is pathogenic to pigs but safe for humans, which can be handled outside a BSL-3 facility. TGEV, like SARS-CoV-2, SARS-CoV, and other coronaviruses, is a member of the coronaviridae family, and shares most structural features with SARS-CoV-2. Both are enveloped, single-stranded positive sense RNA viruses. Both virions are approximately spherical particles of about 100–160 nm in diameter and contain 4 structural proteins, spike (S), envelope (E), membrane (M), and nucleocapsid (N). The S, E, and M are membrane proteins. The N encapsulates the viral genome and packages it into a ribonucleoprotein [29, 30]. The S proteins, which are key to virus binding to the receptor and infecting the host cell, are structurally similar to other coronaviruses [31]. These common features made TGEV and other human coronaviruses indistinguishable in structure and components, and made the TGEV an excellent model for studying sensitivity and stability to antiviral agents [32, 33]. In fact, TGEV has been used as a surrogate for SARS CoV and SARS-CoV-2 for susceptibility testing to biocidal agents and harsh environments [34, 35]. Our comparison will provide a technical reference for the establishments of a thermal inactivation protocol, which could be critical for virus inactivation efficiency, diagnostic accuracy, and biosafety.

## Methods

### Thermal equilibrium assessment

Conical tubes filled with pure water sample were precooled to 8 ℃ and placed in a water bath (Shanghai Yiheng Scientific Instrument, China) or a dry air oven with forced airflow (Memmert, Germany) that were preheated to 56 ℃. The temperatures of samples were probed and recorded continuously at a frequency of every second with a digital thermometer SSN-11E (range: -40 to 125℃/-40 to -257℉; sensitivity: 0.1 ± 0.5℃/0.2 ± 0.5℉, YOWEXA, China). For consistency, the probe was immersed 30 mm below the sample surface and in the center of the tube without contacting the tube wall.

### Virus and cell line

Transmissible gastroenteritis virus (TGEV) and the porcine intestinal columnar epithelial cell line (IPEC-J2) were kindly provided by Dr. Zhiwen Xu, Sichuan Agricultural University, China. IPEC-J2 cells were cultured in Dulbecco’s modified Eagle’s medium (DMEM, Thermo Fisher Scientific, MA) supplemented with 10% fetal bovine serum (Thermo Fisher Scientific, MA). Viral stocks were prepared by infecting 80% confluent layers of IPEC-J2 cells with 5 MOI of TGEV in 75 cm^2^ flasks. After incubation at 37℃ for 1 h in 5 mL 2% FBS DMEM medium, the flask was supplemented with 15 mL of 2% FBS DMEM medium and cultured for another 24–48 h. Cells were collected, lysed by freeze-thawing, and spun at 3,000 g for 15 min. Supernatants were collected, aliquoted, and stored at -80℃ as virus stock. Virus titers were determined by the 50% tissue culture infectious doses (TCID50) assay [36]. Briefly, IPEC-J2 cells were seeded into a 96-well plates at 2 × 10^4^ cells/well. After reaching 80% confluence, the wells were infected with 100 µL of ten-fold serially diluted TGEV in eight replicates and cultured at 37 °C for 1 h. The wells were replenished with 100 µL of DMEM medium supplemented with 2% FBS, and incubated for another 48–72 h at 37 °C. The number of cytopathic wells in each dilution was counted. Virus titers were calculated by the Reed-Muench method and expressed as TCID50.

### Virus thermal inactivation

TGEV stocks at 10 × 10^6.16^ TCID50 in 1 mL each were added into 1.5 mL conical microcentrifuge tubes and precooled to 4 °C. The tubes were placed in a preheated water bath at 56℃ or a dry air oven with forced airflow. At the indicated time intervals, the tubes were removed and quenched on ice. Samples were then subject to limited dilution and the virus titers were assessed by plaque assay as described above.

### Statistical analysis

Data were analyzed with GraphPad Prism 6.0 (GraphPad, La Jolla, CA, USA).

## Results

### Water bath has higher heating rates than air oven

**Fig. 1:**
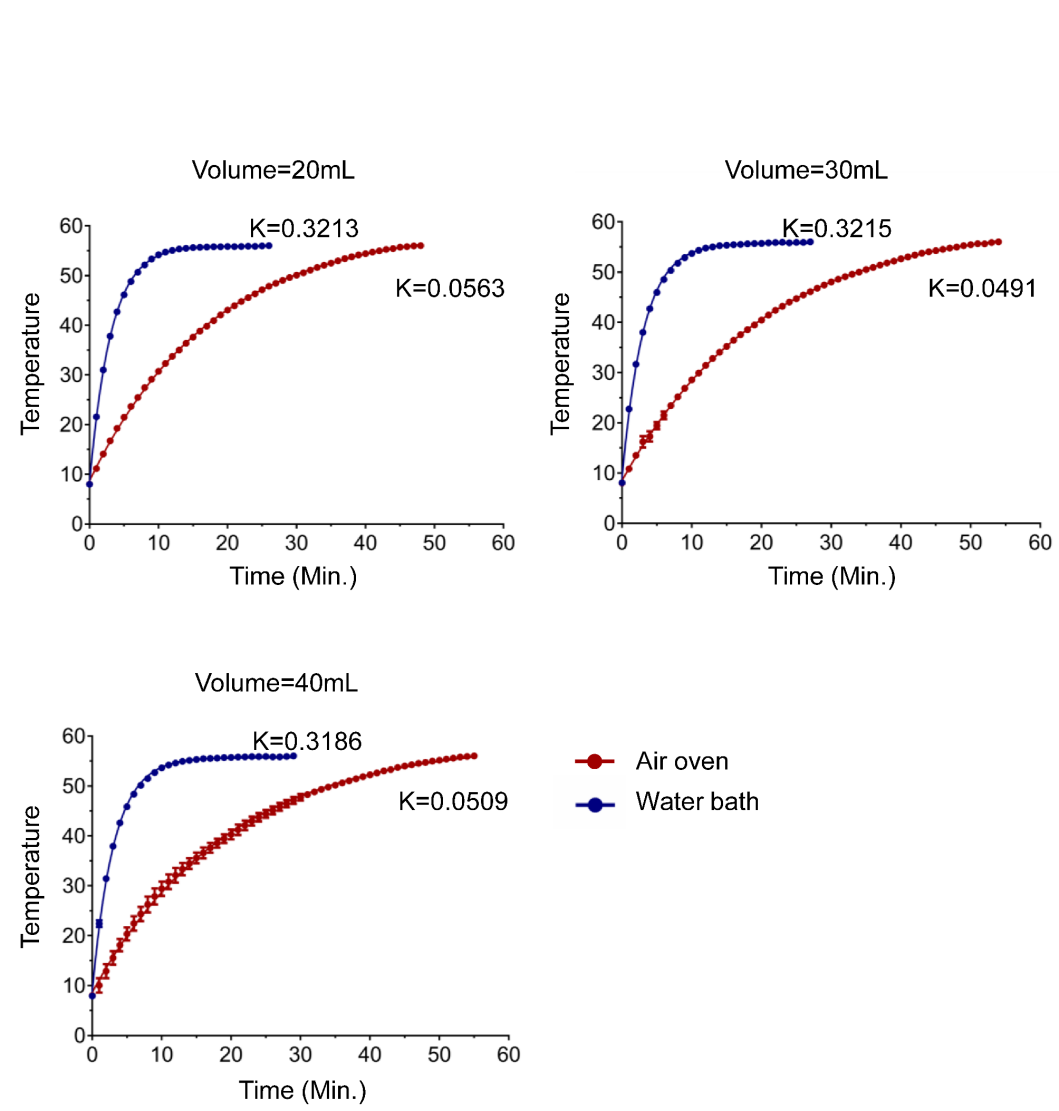
**Heating rates of water bath and air oven.** Pure water samples in conical tubes were precooled to 8 ℃ and placed into water bath and air oven. The temperature was monitored and recorded with a digital thermometer in real time and at a frequency of every second. The heating rate (K) was calculated with Dissociation - One phase Exponential Decay with GraphPad Prism. Data are representative of 3 independent experiments

To compare the heating rates of thermal inactivation devices, pure water samples in 50 mL conical polypropylene tubes were precooled to 8 ℃, loaded on a rack, and placed in a water bath or air oven, which are the most commonly used in hospital and biology laboratories, where the thermal energy is conducted by water or by forced airflow, respectively. The forced airflow increases the air velocity and distributes the heat evenly within the oven. In the water bath, the sample height matched the level of the heating water in the tank. The sample temperature was probed and recorded in real time at a frequency of every second. The dynamics of the temperature equilibrium over time are shown in Fig. 1, and were analyzed with the Dissemination-One Phase Exponential Decay. The K-value represents the heating rates. The result shows that water bath has significantly higher heating rate than air oven, and samples in water bath need less time than those in air oven to reach 56 °C from 8 °C initial temperature.

### Effect of sample volume and container on the heating rate

**Fig. 2:**
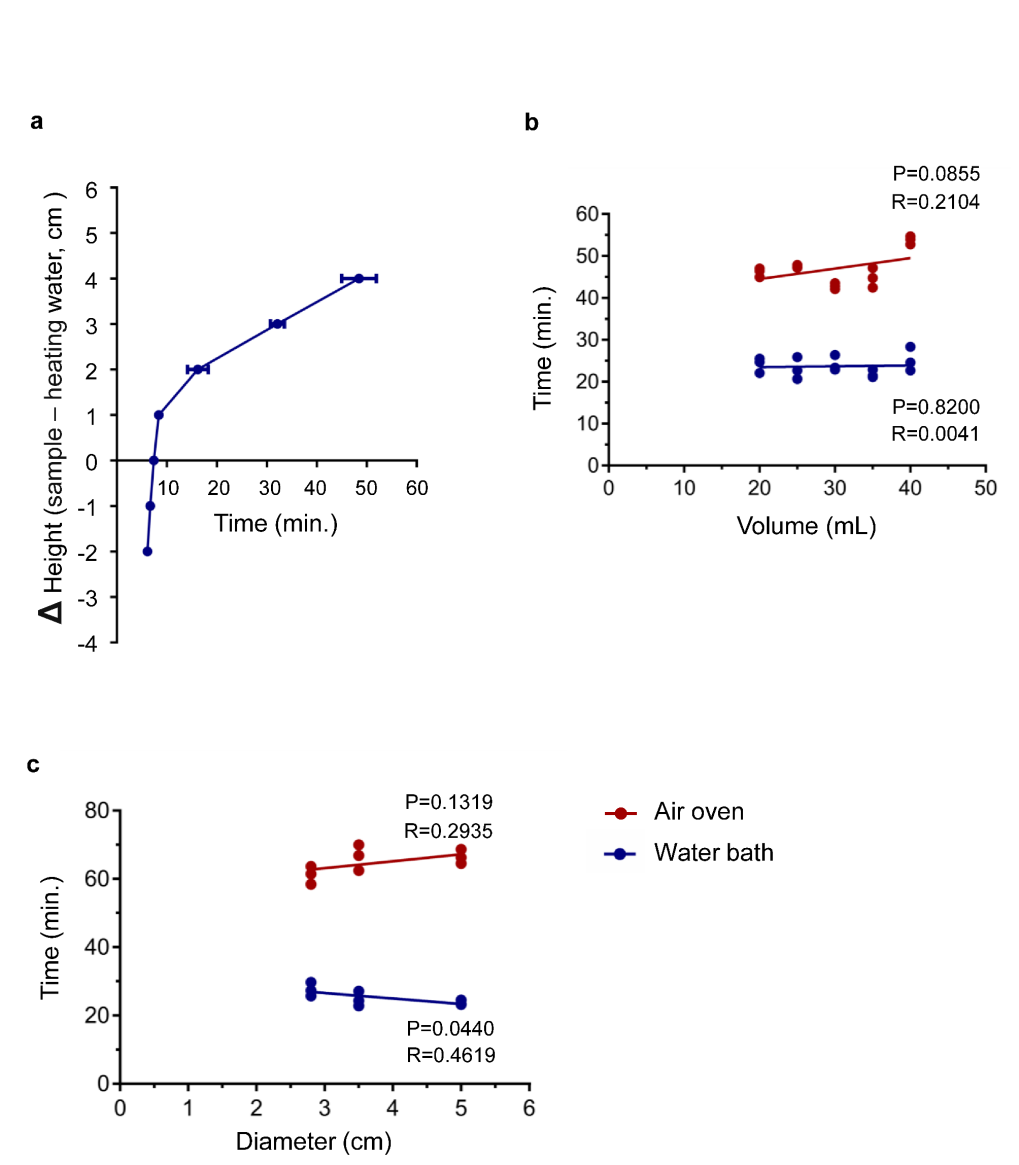
**Effect of sample height, volume, and container diameter on duration to 56** °C. (**a**) Conical tubes filled with 10 mL pure water sample in each were precooled to 8 ℃ and placed in a water bath preheated to 56 ℃. The sample height matched the level of heating water, or kept higher or lower than the level of heating water in scales respectively. The height difference was expressed as Δ. The time lapse of the sample to 56 ℃ was presented as mean ± SD from 3 independent experiments. (**b**) Conical tubes were filled with pure water sample in different volume were precooled to 8 ℃ and placed in water bath and air oven preheated to 56 ℃. The time lapse of water sample to 56 ℃ was shown. (**c**) Containers with indicated diameter were filled with 40 mL of pure water sample and precooled to 8 ℃. After being placed in water bath or air oven preheated to 56 ℃, the time lapse of water sample to 56 ℃ was shown. Data in (b) and (c) were representative of 3 independent experiments and analyzed by linear regression

Sample volume had a different effect on thermal equilibrium in the water bath and air oven. In the water bath, increasing the sample volume but matching the height to the level of the heating water did not increase the time lapse to 56 °C (Fig. 2b). This was consistent with the observation shown in Fig. 1, where the heating rate K was maintained consistent with the increase in sample volume. In the air oven, increasing the sample volume increased the time lapse to 56 °C. The time lapses between 20 mL and 30 mL, 30 mL and 40 mL were significantly different. Details of the time lapse to 56 °C are shown in Table 1.

**Table 1:** Time lapse of specimen to 56 ℃

Sample volume (mL)	20	30	40
Water bath (min.)	24.077 ± 1.789^*^	24.200 ± 1.916^*^	25.193 ± 2.876^*^
Air oven (min.)	46.157 ± 1.055*	52.137 ± 2.290^*/**^	53.793 ± 0.964^*/**^

* p < 0.05 between water bath and air oven in respective volumes; ** p < 0.05 between 20 mL and 30 mL, 20 mL and 40 mL in air oven. (mean ± SD, t-Test)

In water bath inactivation, the sample height may not always match the level of heating water level in routine practice, and the difference would affect the thermal equilibrium. Our study showed that samples with a height above the level of heating water needed extra time to 56 °C, and the extended time correlated with the difference in heights (Δ). As shown in Fig. 2a, the sample with a height higher than the heating water in 40 mm needed an additional 41.07 min to 55 °C compared with the sample with a height matching the heating water, and failed to reach 56 °C as the pre-set heating water temperature even when the duration was extended to more than 85 min.

Increasing the diameter of the sample container increases the distance of heat energy transferred to the center of the sample but increases the contact area with the heating medium if the sample volume is kept constant. We tested containers with diameters of 23, 28, and 45 mm filled with 40 mL of samples, and found that increasing the diameter of the container slightly decreased the time to 56 °C in the water bath, but slightly increased the time to 56 °C in the air oven, although the differences were not significant (Fig. 2c). Samples in each container tested required additional time to reach 56 °C in the air oven compared to the water bath.

### Water bath is more efficient at TGEV inactivation than air oven

**Fig. 3:**
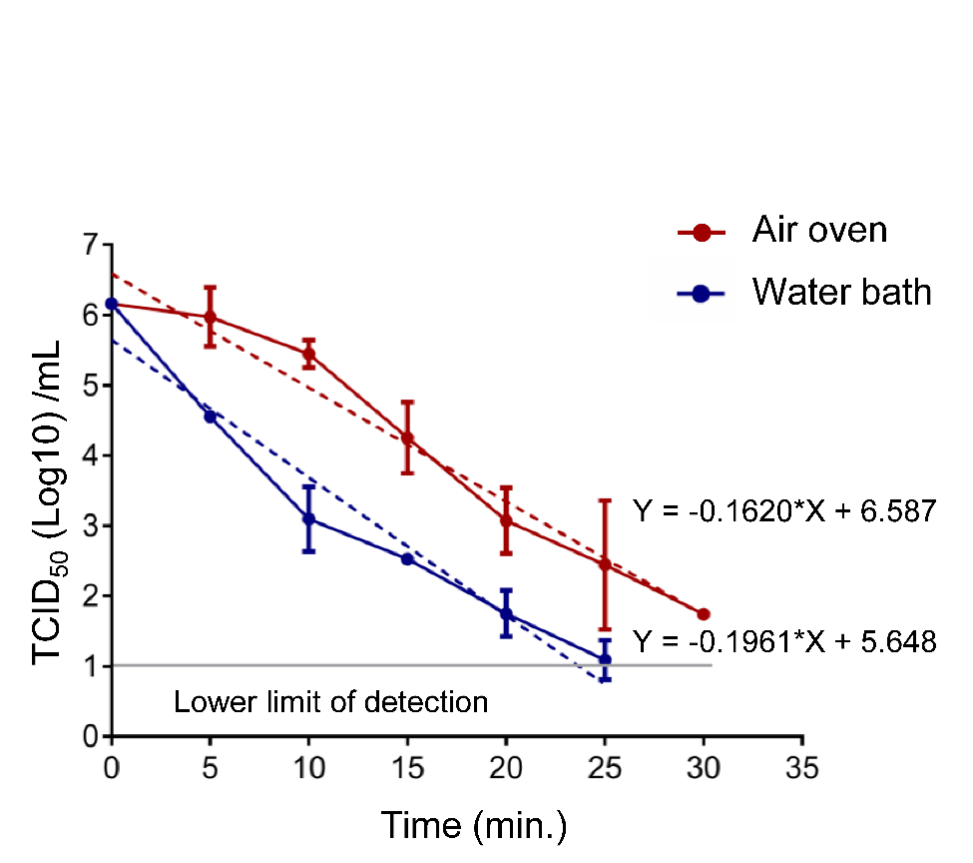
**Efficiency of pathogen inactivation by water bath and air oven.** TGEV in 1 mL was precooled to 8 ℃ and placed into 56 ℃ water bath or air oven respectively, removed at the indicated time intervals, and quenched on ice. Virus titers were assessed and expressed as TCID50. The dot line in blue and red showed the best-fit pattern of water and air oven inactivation by linear regression analysis respectively. The gray line represents the lower limit of detection at 10e1TCID50 /mL. Data are representative of 3 independent experiments and presented as mean ± SD. The low limit of detection for viral cytopathic effect was 10e1 TCID50 /mL

Samples containing TGEV 10e6.16 TCID50 in each tube were placed in a water bath or air oven that were preheated to 56 °C, removed at the indicated time intervals, quenched on ice, and assessed for virus titer. Both inactivation data were analyzed by linear regression curves in a logarithmic plot of virus titer versus inactivation time (Fig. 3). The best-fit line is shown in dot line with a coefficient of -1.961and − 0.162 for water bath and air oven respectively. We found that the virus titer diminished more quickly and significantly in the water bath than in the air oven. A 4 Log10 reduction, representing a 99.99% inactivation, required 17.77 min in the water bath but 27.33 min in the air oven. After 26 min, the virus titer dropped below the detection limit of 10e1TCID50 /mL by water bath inactivation, while the titer remained at 10e2.44 TCID50 /mL by air oven treatment. After 30 min, 10e1.74 TCID50 /mL remained in the air oven inactivated sample.

## Discussion

Our study demonstrates that water bath is more efficient than air oven in thermal inactivation of pathogenic microorganisms, and eliminates the infectivity in a timely and consistent manner. In addition, the water bath reduces the risk of prolonged heat exposure and the spread of pathogens by forced airflow, and is energy efficient. This efficiency is less likely to vary with changes in sample volume, providing convenience and quality assurance in the daily practice of clinical and biological laboratories. The mechanism underlying the efficiency is the advantages of thermal conductivity, specific heat capacity, and heating rate of the water bath over the air oven. Assurance of pathogen inactivation guarantees biosafety; minimization of heat exposure reduces alternation to specimen, especially to critical biomarkers, thus, improving the quality of diagnosis. Water bath is recommended for routine processing of infectious specimens. Our evaluation highlights an often overlooked but critical issue for biosafety and diagnostics, and demonstrates the importance of defining the inactivation device in protocols and guidelines of specimen management.

Incomplete pathogen inactivation compromises biosafety; overheating damages integrity of sample and alters assay results. Prolonged heating has been a temptation to ensure inactivation with low efficiency devices. During the pandemic of COVID-19, thermal inactivation provided a convenient and rapid approach to allow specimens to be processed in BSL-2 facilities. However, evaluation found that heat treatment altered the quantity of SARS-CoV-2 RNA and reduced the sensitivity of pathogen identification, particularly for those with low viral loads [20]. A water bath at 56 °C for 30 min reduced 48.55% of nucleocapsid gene copies and 56.40% of ORF1ab gene copies; an air oven at 80 °C for 20 min reduced 50–60% of SARS-CoV-2 RNA copies. After autoclaving at 121 °C for 20 min or boiling at 100 °C for 20 min, no viral RNA could be detected in the samples [26]. A study found that 56 °C for 30 min or 60 °C for 60 min had little effect on the detection of viral RNA in specimens with viral loads > 6 Log10 TCID50, but reduced the quantity of RNA by at least 4 Log10 TCID50, while 92 °C for 15 min reduced viral RNA to a level below the detection limit of conventional quantitative PCR [21]. Thermal inactivation of sera samples also had a negligible impact on IgG binding capacity [18] and significantly reduced the neutralizing activity against SARS-CoV-2 [21]. Heat treatment at 56 °C for 60 min decreased thyroid-stimulating hormone, aspartate aminotransferase, and pancreatic amylase in plasma by 23–30% compared with no treatment, and damaged creatine kinase, myoglobin, alanine aminotransferase, γ-glutamyl transferase, lactate dehydrogenase, alkaline phosphatase, and the blood coagulation indicators. In contrast, thyroxine was increased by 2.4 folds by 30 min of thermal treatment [37]. The temperature and duration of heating must be minimized to balance pathogen inactivation with the nature of the specimen. Increasing the heating rate can reduce the risk of heating damage and minimize the negative impact on downstream diagnostics without compromising the biosafety.

The mechanism underlying water bath efficiency is the thermal conductivity, specific heat capacity, and heating rate. Thermal energy transfer through water is 20 times faster than that through air, and the specific heat capacity of water is 4.23 times higher than that of air. A high heating rate rapidly denatured nucleic acids and altered protein structure of pathogens [38], leading to a high inactivation efficiency [39]. The heating rate also has a profound effect on host cells harboring viral pathogens. Thermodynamic reaction and physiochemistry showed the development of thermal tolerance below the breakpoint under the slope of the Arrhenius plot [40], and demonstrated that thermal tolerance developed to a greater extent at slow heating rates [41–43]. A high heating rate also resulted in additional sensitization of pathogens to heat thus a quick reduction in infectivity. For a heating rate of 20 °C /minute to 14.7 °C /second, there was a more than 10-fold increase in inactivation efficiency [44]. In contrast, decreasing the heating rate from 10 °C/minute to 2 °C /minute increased heat resistance and resulted in incomplete inactivation [45]. It was hypothesized that a response to heat stress, such as upregulation of heat shock protein, could be induced by slow heating, leading to adaption to heat treatment. Our data also showed that the air oven required approximately 10 min more than the water bath to achieve a 4Log10 reduction in TGEV. Forced air circulation may facilitate the thermal energy exchange between the sample and hot air, but the heat transfer and specific heat capacity limited the heating rate of air oven. In addition, water bath has no forced airflow and circulation, thus, is safer to the environment. Water bath is preferred for routine processing of infectious specimen.

The efficiency of the water bath also ensures a consistent inactivation outcome. In routine clinical practice, especially during the pandemic, specimens were of various volume sizes and in overwhelming quantity. The high workload challenged the processing speed, capacity, and biosafety. The dynamics of thermal equilibria varied with specimens of different volume by air oven treatment, suggesting uncertainty of inactivation outcome. Prolonged hot air treatment was a temptation to ensure biosafety but compromised the integrity of specimen and the downstream diagnostics. This uncertainty can be averted by using a water bath as the preferred method. Our data showed that thermal equilibria did not vary with specimens in different volumes, nor with different types of containers tested. Water bath is preferred for efficient inactivation with consistent inactivation outcome.

Our study has limitations. We used a TGEV model to study the efficiency of sample inactivation by water bath and air oven, and to address concerns about biosafety and diagnostic quality of COVID-19 specimens. We did not evaluate this study using SARS-CoV-2 as a biosafety precaution although both viruses share most of the component and structural features, and TGEV has been used as a model to explore the stability of coronaviruses to harsh treatment. We focused on viral samples in the fluid state, which are the most common in clinical and biological laboratories. Our conclusion may not be applicable to specimen in solid and dry states and inactivated with other devices such as hot steam. Protein, salt, and other components in specimens may affect thermal inactivation, and protect pathogens by reducing the susceptibility to heat. For instance, SARS-CoV survived longer in PBS than in dechlorinated tap water [46]. Proteolytic enzymes increased the susceptibility of enteric viruses to thermal inactivation by cleaving viral proteins and exposing viral RNA [47]. Protein in the sample medium provided protection against SARS-CoV. With 20% protein, infectivity was reduced by less than 2Log10 after 30 min at 56 °C, leaving a residual infectivity. The sample had to be treated at 60 °C for 30 min for complete inactivation [48]. Our conclusion needs to be evaluated in a wide range of applications.

## Conclusions

Heating device has a significant impact on inactivation efficiency and biosafety. Water bath is relatively superior in thermal conductivity, specific heat capacity and heating rate, and confers the efficiency of thermal inactivation of infectious specimen.; Air oven has advantage of capacity and convenience, and can process specimens in bulk but needs prolonged duration relative to water bath for completion of inactivation. In general, water bath offers a balance of convenience, effectiveness, biosafety, environment friendliness, energy conservation, and sample integrity for specimen inactivation, as well as the consistency of inactivation outcomes. The advantages of water bath inactivation may be applicable to the inactivation of other pathogenic microorganisms. Our study supports the proposal of defining the heating device in standard protocols and guidelines for the thermal inactivation of specimens with a defined temperature and duration to ensure the biosafety and accuracy of diagnostics.

## Data Availability

The datasets used and/or analyzed during the current study are available from the corresponding author on reasonable request.

## List of Abbreviations

COVID-19: Corona Virus Disease 2019
SARS-CoV-2: Severe acute respiratory syndrome coronavirus 2
SARS-CoV: Severe acute respiratory syndrome coronavirus
PCR: Polymerase chain reaction
WHO: World Health Organization
CDC: USA Centers for Disease Control and Prevention, USA
TGEV: Transmissible gastroenteritis virus of swine
IPEC-J2: Porcine intestinal columnar epithelial cell line
TCID50: 50% tissue culture infective doses
Δ: The difference in heights
K: The heating rate
